# Bis(benzoyl­acetonato)bis­(1,3-di-4-pyridyl­propane)manganese(II)

**DOI:** 10.1107/S1600536809009891

**Published:** 2009-03-25

**Authors:** Yan Zhou, Wen-Na Zhao, Lei Han

**Affiliations:** a Faculty of Materials Science & Chemical Engineering, Ningbo University, Zhejiang 315211, People’s Republic of China; b Key Laboratory for Molecular Design and Nutrition Engineering of Ningbo, Ningbo Institute of Technology, Zhejiang University, Zhejiang 315100, People’s Republic of China

## Abstract

In the title compound, [Mn(C_10_H_9_O_2_)_2_(C_13_H_14_N_2_)_2_], the Mn^II^ ion lies on a crystallographic inversion center and has a slightly distorted octa­hedral coordination environment. Weak π–π stacking inter­actions, with centroid–centroid distances of 3.862 (2) and 3.887 (5) Å, and significant C—H⋯π inter­actions help to stabilize the crystal structure. The atoms of the unique terminal 4-pyridine­propane group are disordered over two sites, the ratio of refined occpancies being 0.712 (7):0.288 (7).

## Related literature

For the β-diketone group, see: Yoshida *et al.* (1999 ▶). For factors influencing structures and applications, see: Ghosh *et al.* (2004 ▶). For the 1-benzoyl­acetone ligand, see: Han & Zhou (2008 ▶); Bučar & Meštrović (2003 ▶); Meštrović *et al.* (2004 ▶). For 1,3-bis(4-pyridyl)propane, see: Carlucci *et al.* (2002 ▶); Han *et al.* (2007 ▶).
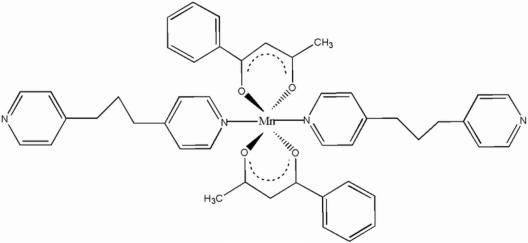


## Experimental

### 

#### Crystal data

[Mn(C_10_H_9_O_2_)_2_(C_13_H_14_N_2_)_2_]
*M* *_r_* = 773.81
Triclinic, 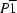

*a* = 9.771 (2) Å
*b* = 10.269 (2) Å
*c* = 10.485 (2) Å
α = 79.84 (3)°
β = 77.68 (3)°
γ = 89.45 (3)°
*V* = 1011.3 (3) Å^3^
*Z* = 1
Mo *K*α radiation
μ = 0.37 mm^−1^
*T* = 298 K
0.43 × 0.27 × 0.14 mm

#### Data collection

Rigaku R-AXIS RAPID diffractometer
Absorption correction: multi-scan (*ABSCOR*; Higashi, 1995 ▶) *T*_min_ = 0.886, *T*_max_ = 0.949
9996 measured reflections
4583 independent reflections
2625 reflections with *I* > 2σ(*I*)
*R*_int_ = 0.038

#### Refinement

*R*[*F* ^2^ > 2σ(*F* ^2^)] = 0.054
*wR*(*F* ^2^) = 0.131
*S* = 1.11
4583 reflections
279 parameters
22 restraints
H-atom parameters constrained
Δρ_max_ = 0.44 e Å^−3^
Δρ_min_ = −0.85 e Å^−3^

### 

Data collection: *RAPID-AUTO* (Rigaku, 1998 ▶); cell refinement: *RAPID-AUTO*; data reduction: *CrystalStructure* (Rigaku/MSC, 2004 ▶); program(s) used to solve structure: *SHELXS97* (Sheldrick, 2008 ▶); program(s) used to refine structure: *SHELXL97* (Sheldrick, 2008 ▶); molecular graphics: *ORTEPII* (Johnson, 1976 ▶); software used to prepare material for publication: *SHELXL97* and *PLATON* (Spek, 2009 ▶).

## Supplementary Material

Crystal structure: contains datablocks I, global. DOI: 10.1107/S1600536809009891/lh2780sup1.cif

Structure factors: contains datablocks I. DOI: 10.1107/S1600536809009891/lh2780Isup2.hkl

Additional supplementary materials:  crystallographic information; 3D view; checkCIF report

## Figures and Tables

**Table:** 

Mn1—O2	2.124 (2)
Mn1—O1	2.157 (2)
Mn1—N1	2.330 (3)

**Table:** 

O2—Mn1—O2^i^	180
O2—Mn1—O1^i^	97.35 (8)
O2—Mn1—O1	82.65 (8)
O1^i^—Mn1—O1	180
O2—Mn1—N1	90.12 (9)
O1—Mn1—N1	91.32 (9)
O2—Mn1—N1^i^	89.88 (9)
O1—Mn1—N1^i^	88.68 (9)
N1—Mn1—N1^i^	180

Symmetry code: (i) 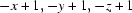.

**Table 2:** Hydrogen-bond geometry (Å, °)

*D*—H⋯*A*	*D*—H	H⋯*A*	*D*⋯*A*	*D*—H⋯*A*
C11—H11*A*⋯*Cg*1	0.93	2.56	3.159 (4)	122
C14—H14*A*⋯*Cg*2^ii^	0.93	2.91	3.738 (5)	149
C14—H14*A*⋯*Cg*3^ii^	0.93	2.63	3.440 (9)	147
C15—H15*A*⋯*Cg*1	0.93	2.60	3.206 (3)	123
C20—H20*A*⋯*Cg*4^iii^	0.93	2.65	3.529 (7)	158

Symmetry codes: (ii) 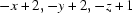; (iii) 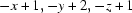.*Cg*1, *Cg*2, *Cg*3 and *Cg*4 are the centroids of the Mn1/O1/C1–C3/O2, N2/C19–C23, N2*A*/C19*A*–C23*A* and C4–C9 rings, respectively.

**Fig. 1.**
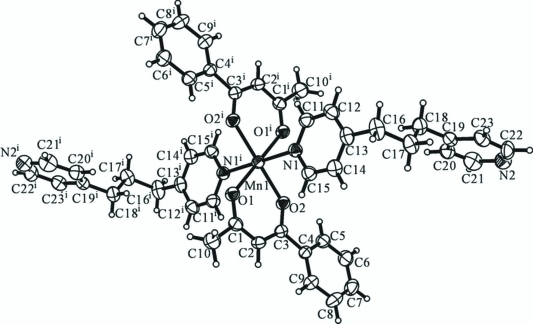
The molecular structure of (1) with 30% probability ellipsoids. The minor component disorder atoms have been removed for clarity (Symmetry codes (i): 1-x, 1-y, 1-z).

**Fig. 2.**
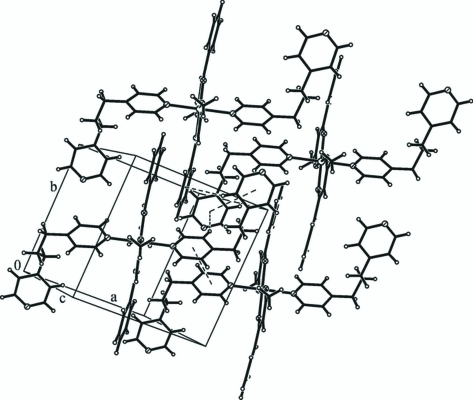
Part of the crystal structure of (1), showing π···π stacking interactions and C—H···π interactions as dashed lines. The minor component disorder atoms have been removed for clarity.

## supplementary crystallographic information

### Comment

Great attention has been given to the β-diketone group, as it can chelate divalent 3d-electron metal elements with a heterocyclic base as an electron donor and a number of complexes have been reported in the literature (Yoshida *et al.*, 1999). Many factors, such as guests with different shapes and sizes, the shape of counterions, metal ions and nonconcovalent inter- or intramolecular forces (e.g. hydrogen bonding, π···π stacking and C—H···π interactions) play important roles in determining their structures and applications (Ghosh *et al.*, 2004). 1-Benzoylacetone (Hbzac) is an excellent choice of ligand, not only due to its chelating coordinating effect to the metal center, but also to its ability to act as an anionic ligand to balance the charge and form a neutral framework (Han & Zhou, 2008; Bučar *et al.*, 2003; Meštrović *et al.*, 2004). Another organic ligand, 1,3-bis(4-pyridyl)propane) (bpp), is a long and flexible multi-functional linker, which can adopt different conformations with respect to the relative orientations of the CH_2_ groups (Han *et al.*, 2007; Carlucci *et al.*, 2002). Recently, we synthesized a neutral monomer, [Mn(bzac)_2_(bpp)_2_] through the ambient evaporation of a mixed solution, of which weak π···π stacking and significant C—H···πinteractions are observed in the crystal structure.

There title compound, [Mn(bzac)_2_(bpp)_2_] (1), is centrosymmetric with the Mn^II^ ion adopting a slightly distorted octahedral coordination geometry. As shown in Fig. 1, the asymmetric unit consists of one-half of the molecule. The Mn^II^ ion is coordinated by four O atoms from two symmetry realted bzac anionic ligands in the equatorial plane and two N atoms from two symmetry realted bpp ligands in the axial sites. The chelate ring (Mn/O1/C1/C2/C3/O2) is essentially planar and forms a dihedral angle of 84.96 (8)° with the N1/C11-C15 ring and an angle of 12.49 (9)° with the C4-C9 ring. In the crystal structure there are weak π···π interactions between symmetry related (N1/C11-C15) pyridine rings (symmetry code: 2-x,1-y,1-z) with a centroid-to-centroid distance of 3.862 (2) Å and a perpendicular distance of 3.536 (2)Å and between symmetry related  N2/C19-C23 rings (symmetry code: 2-x,2-y,2-z) with a centroid-to-centroid distance of 3.887 (5)Å and a perpendicular distance of 3.280 (3)Å (see Fig .2). In addition, significant C—H···π interactions (Spek, 2009) (Table 2) help stabilize the crystal structure.

### Experimental

A mixture of 1-benzoylacetone (0.0358 g, 0.2 mmol) and 1,3-bis(4-pyridyl)propane (0.0830 g, 0.4 mmol) in mixed solution of CH_3_CN (10ml) and H_2_O (10ml) was stirred for 30 min. Then MnCl_2_.4H_2_O (0.1547g, 0.8 mmol) was added to the solution and stirred for 1 h. The mixed solution was allowed to stand at room temperature for 15 days. A quantity of yellow block-shaped crystals were obtained and collected by filtration with 20% yield based on MnCl_2_.4H_2_O.

### Refinement

All H atoms on C atoms were positioned geometrically and allowed to ride on their respective parent atoms, with C—H = 0.93 Å and *U**_iso_*(H) = 1.2*U**_eq_*(C) for phenyl and pyridyl H atoms, C—H = 0.96 Å and *U**_iso_*(H) =1.5*U**_eq_*(C) for methyl, C—H = 0.97 Å and *U**_iso_*(H) = 1.2*U**_eq_*(C) for methylene. The atoms of the unique terminal 4-pyridinepropane group are disordered over two sites with a ratio of refined occpancies being 0.712 (7):0.288 (7). The atoms of the minor component of disorder were reined with isotropic displacement parameters.

### Crystal data

**Table:**

[Mn(C_10_H_9_O_2_)_2_(C_13_H_14_N_2_)_2_]	*Z* = 1
*M**_r_* = 773.81	*F*(000) = 407
Triclinic, *P*1	*D*_x_ = 1.271 Mg m^−^^3^
Hall symbol: -P 1	Mo *K*α radiation, λ = 0.71073 Å
*a* = 9.771 (2) Å	Cell parameters from 9996 reflections
*b* = 10.269 (2) Å	θ = 3.1–27.4°
*c* = 10.485 (2) Å	µ = 0.37 mm^−^^1^
α = 79.84 (3)°	*T* = 298 K
β = 77.68 (3)°	Block, yellow
γ = 89.45 (3)°	0.43 × 0.27 × 0.14 mm
*V* = 1011.3 (3) Å^3^	

### Data collection

**Table:**

Rigaku R-AXIS RAPID diffractometer	4583 independent reflections
Radiation source: fine-focus sealed tube	2625 reflections with *I* > 2σ(*I*)
graphite	*R*_int_ = 0.038
Detector resolution: 0 pixels mm^-1^	θ_max_ = 27.4°, θ_min_ = 3.1°
ω scans	*h* = −12→11
Absorption correction: multi-scan (*ABSCOR*; Higashi, 1995)	*k* = −13→13
*T*_min_ = 0.886, *T*_max_ = 0.949	*l* = −13→13
9996 measured reflections	

### Refinement

**Table:**

Refinement on *F*^2^	Primary atom site location: structure-invariant direct methods
Least-squares matrix: full	Secondary atom site location: difference Fourier map
*R*[*F*^2^ > 2σ(*F*^2^)] = 0.054	Hydrogen site location: inferred from neighbouring sites
*wR*(*F*^2^) = 0.131	H-atom parameters constrained
*S* = 1.11	*w* = 1/[σ^2^(*F*_o_^2^) + (0.0212*P*)^2^ + 0.8043*P*] where *P* = (*F*_o_^2^ + 2*F*_c_^2^)/3
4583 reflections	(Δ/σ)_max_ < 0.001
279 parameters	Δρ_max_ = 0.44 e Å^−^^3^
22 restraints	Δρ_min_ = −0.85 e Å^−^^3^

### Special details

**Table:**

Geometry. All esds (except the esd in the dihedral angle between two l.s. planes) are estimated using the full covariance matrix. The cell esds are taken into account individually in the estimation of esds in distances, angles and torsion angles; correlations between esds in cell parameters are only used when they are defined by crystal symmetry. An approximate (isotropic) treatment of cell esds is used for estimating esds involving l.s. planes.
Refinement. Refinement of F^2^ against ALL reflections. The weighted R-factor wR and goodness of fit S are based on F^2^, conventional R-factors R are based on F, with F set to zero for negative F^2^. The threshold expression of F^2^ > 2sigma(F^2^) is used only for calculating R-factors(gt) etc. and is not relevant to the choice of reflections for refinement. R-factors based on F^2^ are statistically about twice as large as those based on F, and R- factors based on ALL data will be even larger.

### Fractional atomic coordinates and isotropic or equivalent isotropic displacement parameters (Å^2^)

**Table:**

	*x*	*y*	*z*	*U*_iso_*/*U*_eq_	Occ. (<1)
Mn1	0.5000	0.5000	0.5000	0.0515 (2)	
O1	0.5977 (2)	0.4870 (2)	0.2980 (2)	0.0588 (6)	
O2	0.4772 (2)	0.70055 (19)	0.4153 (2)	0.0583 (6)	
N1	0.7142 (3)	0.5550 (2)	0.5435 (3)	0.0554 (7)	
C1	0.6164 (3)	0.5749 (3)	0.1942 (3)	0.0561 (8)	
C2	0.5774 (3)	0.7069 (3)	0.1891 (3)	0.0543 (8)	
H2A	0.5942	0.7611	0.1060	0.065*	
C3	0.5159 (3)	0.7642 (3)	0.2973 (3)	0.0497 (7)	
C4	0.4914 (3)	0.9105 (3)	0.2811 (3)	0.0512 (7)	
C5	0.4131 (4)	0.9594 (3)	0.3870 (4)	0.0663 (9)	
H5A	0.3739	0.9010	0.4644	0.080*	
C6	0.3917 (4)	1.0938 (4)	0.3806 (4)	0.0818 (11)	
H6A	0.3375	1.1247	0.4528	0.098*	
C7	0.4501 (5)	1.1811 (4)	0.2680 (5)	0.0850 (12)	
H7A	0.4377	1.2715	0.2640	0.102*	
C8	0.5270 (4)	1.1340 (4)	0.1614 (4)	0.0819 (12)	
H8A	0.5656	1.1929	0.0841	0.098*	
C9	0.5481 (3)	0.9997 (3)	0.1673 (4)	0.0649 (9)	
H9A	0.6009	0.9693	0.0942	0.078*	
C10	0.6872 (5)	0.5313 (4)	0.0665 (3)	0.0889 (13)	
H10A	0.7800	0.5030	0.0723	0.133*	
H10B	0.6927	0.6039	−0.0061	0.133*	
H10C	0.6340	0.4591	0.0522	0.133*	
C11	0.7529 (4)	0.5049 (3)	0.6567 (3)	0.0638 (9)	
H11A	0.6941	0.4413	0.7173	0.077*	
C12	0.8743 (4)	0.5418 (4)	0.6887 (4)	0.0677 (9)	
H12A	0.8959	0.5029	0.7688	0.081*	
C13	0.9645 (3)	0.6367 (4)	0.6023 (4)	0.0631 (9)	
C14	0.9256 (3)	0.6873 (3)	0.4842 (4)	0.0655 (9)	
H14A	0.9830	0.7503	0.4215	0.079*	
C15	0.8025 (3)	0.6449 (3)	0.4595 (3)	0.0610 (8)	
H15A	0.7793	0.6812	0.3793	0.073*	
C16	1.0969 (4)	0.6845 (4)	0.6333 (4)	0.0850 (12)	
H16A	1.1748	0.6787	0.5599	0.102*	0.712 (7)
H16B	1.1152	0.6272	0.7115	0.102*	0.712 (7)
C17	1.0873 (5)	0.8315 (6)	0.6584 (6)	0.0735 (18)	0.712 (7)
H17A	1.1786	0.8627	0.6649	0.088*	0.712 (7)
H17B	1.0597	0.8880	0.5843	0.088*	0.712 (7)
C18	0.9821 (5)	0.8393 (5)	0.7845 (5)	0.0715 (18)	0.712 (7)
H18A	0.8921	0.8054	0.7778	0.086*	0.712 (7)
H18B	1.0112	0.7826	0.8578	0.086*	0.712 (7)
C19	0.9642 (5)	0.9769 (5)	0.8155 (6)	0.0585 (14)	0.712 (7)
C20	0.8366 (7)	1.0339 (8)	0.8254 (7)	0.068 (2)	0.712 (7)
H20A	0.7619	0.9881	0.8099	0.082*	0.712 (7)
C21	0.8167 (10)	1.1545 (10)	0.8570 (9)	0.091 (4)	0.712 (7)
H21A	0.7279	1.1890	0.8605	0.109*	0.712 (7)
C22	1.0392 (10)	1.1755 (10)	0.8710 (12)	0.084 (3)	0.712 (7)
H22A	1.1114	1.2260	0.8848	0.101*	0.712 (7)
C23	1.0715 (6)	1.0503 (7)	0.8387 (7)	0.0711 (19)	0.712 (7)
H23A	1.1614	1.0180	0.8331	0.085*	0.712 (7)
N2	0.9127 (8)	1.2275 (7)	0.8834 (7)	0.083 (3)*	0.712 (7)
H16C	1.1545	0.7357	0.5537	0.102*	0.288 (7)
H16D	1.1497	0.6092	0.6635	0.102*	0.288 (7)
C17A	1.0611 (15)	0.7711 (11)	0.7420 (13)	0.067 (4)*	0.288 (7)
H17C	0.9851	0.7311	0.8133	0.080*	0.288 (7)
H17D	1.1421	0.7844	0.7785	0.080*	0.288 (7)
C18A	1.0178 (15)	0.9009 (11)	0.6687 (12)	0.067 (4)*	0.288 (7)
H18C	1.0926	0.9332	0.5927	0.081*	0.288 (7)
H18D	0.9355	0.8841	0.6355	0.081*	0.288 (7)
C19A	0.9859 (14)	1.0068 (12)	0.7515 (15)	0.059 (4)*	0.288 (7)
C20A	0.8553 (17)	1.0579 (18)	0.7869 (19)	0.065 (6)*	0.288 (7)
H20B	0.7773	1.0231	0.7654	0.079*	0.288 (7)
C21A	0.843 (2)	1.1620 (17)	0.855 (2)	0.054 (6)*	0.288 (7)
H21B	0.7535	1.1924	0.8812	0.064*	0.288 (7)
C22A	1.071 (2)	1.170 (2)	0.861 (3)	0.061 (6)*	0.288 (7)
H22B	1.1456	1.1995	0.8918	0.073*	0.288 (7)
C23A	1.0905 (17)	1.0689 (16)	0.7889 (16)	0.065 (6)*	0.288 (7)
H23B	1.1814	1.0408	0.7635	0.078*	0.288 (7)
N2A	0.9475 (18)	1.2239 (16)	0.8872 (17)	0.066 (5)*	0.288 (7)

### Atomic displacement parameters (Å^2^)

**Table:**

	*U*^11^	*U*^22^	*U*^33^	*U*^12^	*U*^13^	*U*^23^
Mn1	0.0558 (4)	0.0467 (4)	0.0503 (4)	0.0015 (3)	−0.0082 (3)	−0.0078 (3)
O1	0.0646 (14)	0.0521 (13)	0.0569 (14)	0.0078 (11)	−0.0061 (11)	−0.0110 (11)
O2	0.0687 (14)	0.0483 (12)	0.0537 (13)	0.0068 (10)	−0.0045 (11)	−0.0088 (10)
N1	0.0563 (16)	0.0563 (16)	0.0534 (16)	0.0028 (13)	−0.0106 (13)	−0.0110 (13)
C1	0.0542 (19)	0.063 (2)	0.0501 (19)	0.0049 (16)	−0.0084 (15)	−0.0119 (16)
C2	0.0583 (19)	0.0516 (18)	0.0499 (18)	0.0080 (15)	−0.0104 (15)	−0.0028 (15)
C3	0.0432 (17)	0.0510 (17)	0.0534 (19)	−0.0011 (14)	−0.0101 (14)	−0.0055 (15)
C4	0.0473 (17)	0.0473 (17)	0.0599 (19)	0.0012 (14)	−0.0172 (15)	−0.0051 (15)
C5	0.076 (2)	0.058 (2)	0.066 (2)	0.0128 (18)	−0.0185 (19)	−0.0120 (17)
C6	0.104 (3)	0.064 (2)	0.086 (3)	0.026 (2)	−0.030 (2)	−0.026 (2)
C7	0.101 (3)	0.053 (2)	0.111 (3)	0.011 (2)	−0.041 (3)	−0.017 (2)
C8	0.087 (3)	0.052 (2)	0.099 (3)	0.000 (2)	−0.019 (2)	0.007 (2)
C9	0.063 (2)	0.055 (2)	0.072 (2)	0.0001 (16)	−0.0108 (18)	−0.0048 (17)
C10	0.119 (3)	0.082 (3)	0.059 (2)	0.014 (2)	0.003 (2)	−0.022 (2)
C11	0.069 (2)	0.061 (2)	0.059 (2)	0.0008 (17)	−0.0101 (18)	−0.0083 (17)
C12	0.071 (2)	0.075 (2)	0.064 (2)	0.015 (2)	−0.0243 (19)	−0.0189 (19)
C13	0.0507 (19)	0.073 (2)	0.074 (2)	0.0129 (17)	−0.0137 (18)	−0.036 (2)
C14	0.054 (2)	0.074 (2)	0.067 (2)	−0.0038 (17)	−0.0042 (17)	−0.0181 (18)
C15	0.060 (2)	0.066 (2)	0.055 (2)	0.0002 (17)	−0.0079 (17)	−0.0105 (17)
C16	0.060 (2)	0.101 (3)	0.110 (3)	0.016 (2)	−0.025 (2)	−0.056 (3)
C17	0.047 (3)	0.102 (5)	0.075 (4)	−0.007 (3)	−0.007 (3)	−0.030 (4)
C18	0.069 (3)	0.077 (4)	0.070 (4)	−0.005 (3)	−0.009 (3)	−0.023 (3)
C19	0.055 (3)	0.071 (3)	0.051 (3)	−0.002 (3)	−0.013 (3)	−0.011 (3)
C20	0.051 (3)	0.081 (5)	0.074 (5)	−0.003 (3)	−0.019 (3)	−0.009 (4)
C21	0.057 (5)	0.117 (8)	0.099 (6)	0.012 (4)	−0.016 (4)	−0.019 (4)
C22	0.078 (7)	0.093 (6)	0.084 (5)	−0.037 (5)	−0.017 (5)	−0.017 (4)
C23	0.049 (3)	0.096 (5)	0.070 (5)	−0.001 (3)	−0.017 (3)	−0.014 (4)
C16A	0.060 (2)	0.101 (3)	0.110 (3)	0.016 (2)	−0.025 (2)	−0.056 (3)

### Geometric parameters (Å, °)

**Table:**

Mn1—O2	2.124 (2)	C15—H15A	0.9300
Mn1—O2^i^	2.124 (2)	C16—C17	1.576 (6)
Mn1—O1^i^	2.157 (2)	C16—H16A	0.9700
Mn1—O1	2.157 (2)	C16—H16B	0.9700
Mn1—N1	2.330 (3)	C17—C18	1.506 (6)
Mn1—N1^i^	2.330 (3)	C17—H17A	0.9700
O1—C1	1.266 (3)	C17—H17B	0.9700
O2—C3	1.273 (3)	C18—C19	1.505 (6)
N1—C15	1.333 (4)	C18—H18A	0.9700
N1—C11	1.337 (4)	C18—H18B	0.9700
C1—C2	1.400 (4)	C19—C20	1.364 (6)
C1—C10	1.511 (4)	C19—C23	1.384 (6)
C2—C3	1.390 (4)	C20—C21	1.339 (8)
C2—H2A	0.9300	C20—H20A	0.9300
C3—C4	1.505 (4)	C21—N2	1.310 (9)
C4—C5	1.377 (4)	C21—H21A	0.9300
C4—C9	1.383 (4)	C22—N2	1.331 (9)
C5—C6	1.386 (5)	C22—C23	1.403 (8)
C5—H5A	0.9300	C22—H22A	0.9300
C6—C7	1.368 (5)	C23—H23A	0.9300
C6—H6A	0.9300	C17A—C18A	1.521 (13)
C7—C8	1.368 (5)	C17A—H17C	0.9700
C7—H7A	0.9300	C17A—H17D	0.9700
C8—C9	1.385 (5)	C18A—C19A	1.498 (13)
C8—H8A	0.9300	C18A—H18C	0.9700
C9—H9A	0.9300	C18A—H18D	0.9700
C10—H10A	0.9600	C19A—C23A	1.369 (13)
C10—H10B	0.9600	C19A—C20A	1.377 (14)
C10—H10C	0.9600	C20A—C21A	1.377 (14)
C11—C12	1.374 (5)	C20A—H20B	0.9300
C11—H11A	0.9300	C21A—N2A	1.339 (15)
C12—C13	1.382 (5)	C21A—H21B	0.9300
C12—H12A	0.9300	C22A—N2A	1.322 (15)
C13—C14	1.384 (5)	C22A—C23A	1.375 (15)
C13—C16	1.506 (5)	C22A—H22B	0.9300
C14—C15	1.374 (4)	C23A—H23B	0.9300
C14—H14A	0.9300		
			
O2—Mn1—O2^i^	180	C13—C14—H14A	119.9
O2—Mn1—O1^i^	97.35 (8)	N1—C15—C14	123.9 (3)
O2^i^—Mn1—O1^i^	82.65 (8)	N1—C15—H15A	118.1
O2—Mn1—O1	82.65 (8)	C14—C15—H15A	118.1
O2^i^—Mn1—O1	97.35 (8)	C13—C16—C17	112.3 (3)
O1^i^—Mn1—O1	180	C13—C16—H16A	109.1
O2—Mn1—N1	90.12 (9)	C17—C16—H16A	109.1
O2^i^—Mn1—N1	89.88 (9)	C13—C16—H16B	109.1
O1^i^—Mn1—N1	88.68 (9)	C17—C16—H16B	109.1
O1—Mn1—N1	91.32 (9)	H16A—C16—H16B	107.9
O2—Mn1—N1^i^	89.88 (9)	C18—C17—C16	110.2 (4)
O2^i^—Mn1—N1^i^	90.12 (9)	C18—C17—H17A	109.6
O1^i^—Mn1—N1^i^	91.32 (9)	C16—C17—H17A	109.6
O1—Mn1—N1^i^	88.68 (9)	C18—C17—H17B	109.6
N1—Mn1—N1^i^	180	C16—C17—H17B	109.6
C1—O1—Mn1	129.9 (2)	H17A—C17—H17B	108.1
C3—O2—Mn1	131.8 (2)	C19—C18—C17	114.0 (4)
C15—N1—C11	115.8 (3)	C19—C18—H18A	108.7
C15—N1—Mn1	121.5 (2)	C17—C18—H18A	108.7
C11—N1—Mn1	122.6 (2)	C19—C18—H18B	108.7
O1—C1—C2	125.5 (3)	C17—C18—H18B	108.7
O1—C1—C10	116.2 (3)	H18A—C18—H18B	107.6
C2—C1—C10	118.3 (3)	C20—C19—C23	116.6 (5)
C3—C2—C1	125.7 (3)	C20—C19—C18	120.1 (5)
C3—C2—H2A	117.2	C23—C19—C18	123.2 (5)
C1—C2—H2A	117.2	C21—C20—C19	121.3 (7)
O2—C3—C2	124.3 (3)	C21—C20—H20A	119.3
O2—C3—C4	114.9 (3)	C19—C20—H20A	119.3
C2—C3—C4	120.8 (3)	N2—C21—C20	125.0 (9)
C5—C4—C9	118.0 (3)	N2—C21—H21A	117.5
C5—C4—C3	118.4 (3)	C20—C21—H21A	117.5
C9—C4—C3	123.5 (3)	N2—C22—C23	124.8 (7)
C4—C5—C6	121.3 (3)	N2—C22—H22A	117.6
C4—C5—H5A	119.3	C23—C22—H22A	117.6
C6—C5—H5A	119.3	C19—C23—C22	117.3 (6)
C7—C6—C5	120.0 (4)	C19—C23—H23A	121.3
C7—C6—H6A	120.0	C22—C23—H23A	121.3
C5—C6—H6A	120.0	C21—N2—C22	114.8 (7)
C6—C7—C8	119.4 (4)	C18A—C17A—H17C	111.1
C6—C7—H7A	120.3	C18A—C17A—H17D	111.1
C8—C7—H7A	120.3	H17C—C17A—H17D	109.0
C7—C8—C9	120.7 (4)	C19A—C18A—C17A	114.2 (10)
C7—C8—H8A	119.7	C19A—C18A—H18C	108.7
C9—C8—H8A	119.7	C17A—C18A—H18C	108.7
C4—C9—C8	120.6 (4)	C19A—C18A—H18D	108.7
C4—C9—H9A	119.7	C17A—C18A—H18D	108.7
C8—C9—H9A	119.7	H18C—C18A—H18D	107.6
C1—C10—H10A	109.5	C23A—C19A—C20A	114.3 (12)
C1—C10—H10B	109.5	C23A—C19A—C18A	121.1 (12)
H10A—C10—H10B	109.5	C20A—C19A—C18A	124.4 (12)
C1—C10—H10C	109.5	C21A—C20A—C19A	118.1 (15)
H10A—C10—H10C	109.5	C21A—C20A—H20B	120.9
H10B—C10—H10C	109.5	C19A—C20A—H20B	120.9
N1—C11—C12	123.8 (3)	N2A—C21A—C20A	126.7 (16)
N1—C11—H11A	118.1	N2A—C21A—H21B	116.7
C12—C11—H11A	118.1	C20A—C21A—H21B	116.7
C11—C12—C13	120.2 (3)	N2A—C22A—C23A	120.3 (17)
C11—C12—H12A	119.9	N2A—C22A—H22B	119.9
C13—C12—H12A	119.9	C23A—C22A—H22B	119.9
C12—C13—C14	116.0 (3)	C19A—C23A—C22A	125.0 (15)
C12—C13—C16	122.8 (4)	C19A—C23A—H23B	117.5
C14—C13—C16	121.2 (4)	C22A—C23A—H23B	117.5
C15—C14—C13	120.2 (3)	C22A—N2A—C21A	115.1 (14)
C15—C14—H14A	119.9		

Symmetry codes: (i) −*x*+1, −*y*+1, −*z*+1.

### Hydrogen-bond geometry (Å, °)

**Table:**

*D*—H···*A*	*D*—H	H···*A*	*D*···*A*	*D*—H···*A*
C11—H11A···Cg1	0.93	2.56	3.159 (4)	122
C14—H14A···Cg2^ii^	0.93	2.91	3.738 (5)	149
C14—H14A···Cg3^ii^	0.93	2.63	3.440 (9)	147
C15—H15A···Cg1	0.93	2.60	3.206 (3)	123
C20—H20A···Cg4^iii^	0.93	2.65	3.529 (7)	158

Symmetry codes:  (ii) −*x*+2, −*y*+2, −*z*+1; (iii) −*x*+1, −*y*+2, −*z*+1.
